# The interplay between persistent pathogen infections with tumor microenvironment and immunotherapy in cancer

**DOI:** 10.1002/cam4.70154

**Published:** 2024-09-06

**Authors:** Si Chen, Caihong Yao, Na Tian, Chunying Zhang, Yuemei Chen, Xuting Wang, Yue Jiang, Tonghao Zhang, Tingting Zeng, Yali Song

**Affiliations:** ^1^ Department of Laboratory Medicine West China Hospital, Sichuan University; Sichuan Clinical Research Center for Laboratory Medicine; Clinical Laboratory Medicine Research Center of West China Hospital Chengdu People's Republic of China; ^2^ Anesthesiology Department Qingdao Eighth People's Hospital Qingdao People's Republic of China; ^3^ Department of Statistics University of Virginia Charlottesville Virginia USA

**Keywords:** cancer, chronic infections, immunotherapy, tumor microenvironment

## Abstract

**Background:**

Chronic infections by pathogenic microorganisms play a significant role in cancer development, disrupting the body's immune system and microenvironment. This interference impairs the body's ability to eliminate these microorganisms promptly, allowing them to persist by evading immune defenses.

**Aims:**

This study aimed to explore how chronic pathogenic infections influence the immune microenvironment, impacting tumorigenesis, cancer progression, and treatment strategies. Additionally, it seeks to investigate the effects of these infections on specific immune checkpoints and identify potential targets for immunotherapy.

**Methods:**

We conducted searches, readings, and detailed analyses of key terms in databases like PubMed and Web of Science to evaluate the impact of chronic infections by pathogenic microorganisms on the immune microenvironment.

**Results:**

Our analysis demonstrates a significant association between persistent chronic infections by pathogenic microorganisms and tumorigenesis. Notable impacts on the immune microenvironment include changes in immune cell function and the regulation of immune checkpoints, offering insights into potential targets for cancer immunotherapy.

**Discussion:**

This study highlights the complex relationship between chronic infections and cancer development, presenting new opportunities for cancer immunotherapy by understanding their effects on the immune microenvironment. The influence of these infections on immune checkpoints emphasizes the crucial role of the immune system in cancer treatment.

**Conclusion:**

Chronic infections by pathogenic microorganisms greatly affect the immune microenvironment, tumorigenesis, and cancer treatment. Unraveling the underlying mechanisms can unveil potential targets for immunotherapy, improving our comprehension of the immune response to cancer and potentially leading to more effective cancer treatments in the future.

## 1 INTRODUCTION

Numerous pathogenic microorganisms, encompassing bacteria and viruses, have been linked to cancer development. These pathogens can induce chronic infections in the host post‐infection, with persistence playing a role in tumor progression.^1^ While the body's immune system can effectively manage microorganisms' infections, complete clearance is often unattainable, leading to infection persistence. Microorganisms evade immune responses by influencing immune cells and components associated with inflammation within the tumor microenvironment (TME), posing a significant obstacle to tumor immunotherapy.^2^

This review mainly focuses on carcinogenic pathogens and some newly studies about: Human T‐cell leukemia virus type 1 (HTLV‐1), Human papillomaviruses (HPV), Hepatitis B virus (HBV), Hepatitis C virus (HCV), Epstein–Barr virus (EBV), and Human herpesvirus 8 (KSHV, Kaposi's Sarcoma‐Associated Herpesvirus), Merkel cell polyomavirus (MCPyV), Helicobacter pylori (*H. pylori*).^3^ Moreover, it considers various some carcinogenic pathogens in recent studies, like Fusobacterium nucleatum (*F. nucleatum*), Streptococcus anginosus (*S. anginosus*), Peptostreptococcus anaerobius (*P. anaerobius*), Chlamydia trachomatis (CT), and Mycoplasma. Infections by these agents can lead to various diseases, including cancer (Table 1).

**TABLE 1:** Diseases caused by pathogenic microorganisms associated with cancer.

Pathogen	Type	Associated Diseases	Transmission route
Human T‐cell leukemia virus type 1 (HTLV‐1)	RNA virus	HTLV‐1‐associated myelopathy (HAM), uveitis, chronic inflammatory arthropathy, infectious dermatitis, and leukemia/lymphoma (ATLL)	Bloodborne transmission, sexual contact and mother‐to‐child transmission
Human papillomaviruses (HPV)	DNA virus	Anogenital warts, recurrent respiratory papillomatosis, oropharyngeal carcinoma, and many types of anogenital cancers (including penile, anal, vaginal, vulvar, and cervical cancers) in both men and women	Sexual contact, mother‐to‐child transmission, indirect contact, etc
Hepatitis B virus (HBV)	DNA virus	Acute and chronic hepatitis, cirrhosis and hepatocellular carcinoma (HCC), etc	Blood, blood products, vertical transmission from mother to child, close contact, etc
Hepatitis C virus (HCV)	RNA virus	Chronic hepatitis, cirrhosis and HCC, etc	Sexual contact or mother‐to‐child transmission, etc
Epstein–Barr virus/human gamma herpesvirus 4 (EBV/HHV‐4)	DNA virus	Chronic Active EBV Infection (CAEBV), Infectious Mononucleosis, Burkitt's Lymphoma and Nasopharyngeal Cancer	Saliva transmission
Human Herpesvirus 8/Kaposi's Sarcoma‐Associated Herpesvirus (KSHV/HHV‐8)	DNA virus	Kaposi's sarcoma (KS), primary exudative lymphoma (PEL), and multicentric Castleman's disease (MCD)	Sexual contact, organ transplantation or saliva transmission
Merkel cell polyomavirus (MCPyV)	DNA virus	Mainly associated with Merkel cell carcinoma (MCC)	Sexual, skin‐to‐skin or respiratory droplet transmission
Helicobacter pylori (*H. pylori*)	Gram‐negative bacillus	Closely related to gastritis, peptic ulcer, gastric cancer (GC), and a major causative factor in other types of gastric and extra‐gastric diseases	Fecal‐oral or salivary transmission
Fusobacterium nucleatum	Gram‐negative bacillus	Periodontal disease, acute necrotizing gingivitis, oral cancer, ulcerative colitis, Crohn's disease and colorectal cancer (CRC), etc	Commonly present in the oral cavity, sometimes migrate to the intestines
Streptococcus anginosus	Gram‐positive bacillus	Otitis Media and Sinusitis, induced progressive chronic gastritis, parietal cell atrophy, etc	Droplet transmission (disease transmission from sneezing, coughing etc.)
Peptostreptococcus anaerobius	Gram‐positive anaerobic coccus	Anaerobic bacteria have rarely been reported as the cause of urinary tract infection, CRC	Colonized on gastrointestinal and vaginal mucous
Chlamydia trachomatis	Chlamydia	Conjunctival inflammation, corneal inflammation, urethritis, cervicitis, pneumonia, cervical cancer, etc	Sexual contact, neonatal birth infection, eye‐to‐eye or eye‐to‐hand‐eye contact transmission, etc
Mycoplasma (parasitic bacteria)	Mycoplasma	Often associated with urinary tract infections, infertility and prostate cancer in men	Droplet or sexual contact transmission

## 2 TUMOR MICROENVIRONMENT AND CHRONIC INFECTIONS

The TME predominantly consists of non‐cancerous cellular and non‐cellular elements present within the tumor. Non‐cancer cells primarily include fibrocytes, endothelial cells, neurons, adipocytes, adaptive, and innate immune cells. The non‐cellular components consist mainly of the extracellular matrix (ECM) and soluble products like chemokines, cytokines, growth factors, and extracellular vesicles.^4^ The TME plays a crucial role in the onset and progression of cancer. Various pathogens can cause chronic infections in the host, leading to the persistence of these pathogens in the body, thereby contributing to tumor development.

Over 90% of HTLV‐1 infections are asymptomatic, with only about 5% resulting in adult T‐cell leukemia/lymphoma (ATLL), where the regulatory protein tax plays a pivotal role in persistent viral infection. HPV infection alters the local environment and supports the post‐infectious microenvironment (PIM). Both HBV and HCV exhibit a hepatophilic nature, leading to lifelong chronic infection and viral carriage. EBV enters circulation, causing systemic infection, and remains latent in lymphoid tissues for extended periods. KSHV predominantly infects lymphocytes, with certain genes aiding virus maintenance post‐infection and evasion of host immune responses, facilitating infected cell presence and proliferation. MCPyV, a polyomavirus, infects 60%–80% in childhood, and risk factors such as excessive sunlight exposure, immunosuppression, and advanced age are implicated. *H. pylori* selectively colonizes the gastric epithelium and is found in different regions of the stomach and duodenum. Classified by the World Health Organization (WHO) as a Class I carcinogen associated with gastric cancer (GC) development, *H. pylori* is challenging to eradicate without radical treatment post‐infection, leading to lifelong infection.

Apart from the mentioned pathogens strongly linked to tumorigenesis and progression, additional pathogens have been identified in relation to specific cancers. *F. nucleatum* infection accelerates colorectal cancer (CRC) development by triggers local inflammation within the TME.^5^ *S. anginosus* prompts the occurrence of gastric tumors by interacting with gastric epithelial cells. *P. anaerobius* promotes CRC cell proliferation and induces a pro‐inflammatory microenvironment for tumorigenesis. CT may increase the risk of cervical cancer through infection‐induced inflammation and mycoplasma infection potentially aids in the progression of prostate cancer via chronic inflammation.

Next, we give a detailed introduction to the impact of eight carcinogenic pathogens (seven viruses and one bacterium) that are clearly related to cancers on the TME and immunotherapy. Additionally, this article provides a review of newly studies concerning the relationship between some pathogens with cancer.

## 3 CARCINOGENIC PATHOGENS WITH TME AND IMMUNOTHERAPY

### 3.1 HTLV‐1

HTLV‐1, the first human retrovirus discovered, mostly infects CD4+ T cells, causing a lifelong mostly symptom‐free infection, with only about 5% developing adult T‐cell leukemia/lymphoma (ATLL). ATLL, a type of T‐cell cancer, can be indolent or aggressive.^6^ Diagnosed individuals typically have distinct flower‐like nuclei in peripheral blood and an immunophenotype distinguished by CD3, CD4, and CD25 positivity in T helper cells. Unfortunately, ATLL has poor treatment results, leading to short survival and a bleak outlook.^7^

#### 3.1.1 Effect of HTLV‐1 on the tumor immune microenvironment

ATLL cells are considered to be CD4+ T cells with a Treg phenotype and exhibit strong immunosuppressive activity in vitro. FOXP3+ is a representative marker of Tregs, and FOXP3‐expressing ATLL cells can cause immunosuppression. HTLV‐1 can also undergo immune escape via NK/T cells. The human major histocompatibility complex (MHC) is known as human leukocyte antigen (HLA), and there are three main classes: MHC I, which includes HLA‐1, HLA‐B, and β2microglobulin (β2M), is located on the surface of general cells, MHC II is mostly located on antigen‐presenting cells (APCs), and MHC III mainly encodes complement components. Loss of MHC class I and/or β2M as well as loss of MHC class II can lead to immune escape, with the former by inducing the immune escape of CD8+ Tumor Infiltrating Lymphocytes (TILs), and the latter by protect the body from CD4+ TIL.^8^ Also, the loss of MHC class I and/or β2M is one of the factors influencing the poor prognosis of ATLL. About 33.3% of ATLL expressed MHC class II, and the expression of MHC class II in inert ATLL cells was higher than that in aggressive ATLL cells. MHC class II is considered as an independent good prognostic marker for ATLL.^9^ CD58 activates NK cells and cytotoxic T cells (CTL), and CD58 gene mutation or deletion induces tumor immune escape.^10^ Loss or inactivation of CD58 is more common in invasive ATLL cells.

Immune checkpoint inhibitors, as Programmed Cell Death‐Ligand 1 (PD‐L1)/Programmed Cell Death Protein 1 (PD‐1) axis and Galectin‐9 (GAL9)/ T‐cell Immunoglobulin Mucin‐3 (Tim‐3) pathway both promotes immune escape and T cell deactivation in tumors. In ATLL, PD‐L1 on tumor cells binds to PD‐1 on T cells, suppressing their function. About 27% of ATLL cases have altered PD‐L1 genes,^11^ leading to increased PD‐L1 levels and immune evasion. Additionally, Tim‐3 on ATLL cells and GAL9 on macrophages deactivates T cells and boosts ATLL growth,^12^ and patients with Tim‐3‐positive ATLL respond poorly to chemotherapy.

OX40, also known as Tumor Necrosis Factor Receptor Superfamily Member 4 (TNFRSF4), is primarily expressed on activated T cells. It interacts with its ligand OX40L to stimulate the proliferation, survival, and cytokine production of T cells. ATLL cells frequently express OX40, with the HTLV‐1‐encoded oncoprotein Tax upregulating OX40 gene expression.^13^ OX40L is not only present on APCs like B cells, dendritic cells (DCs), and macrophages, but also on various stromal cells. Moreover, OX40L serves as an independent favorable prognostic marker in ATLL. Its presence on stromal cells can activate ATLL cells expressing OX40. Furthermore, the OX40/OX40L pathway typically suppresses ATLL cells with a regulatory T cell (Treg) phenotype, thereby enhancing the immune cell‐mediated tumor destruction.

In normal T cells, CD28 activity diminishes post T cell activation and is substituted by Cytotoxic T Lymphocyte‐Associated Protein 4 (CTLA‐4) and Inducible Co‐stimulatory Molecules (ICOS) that form a complex with CTLA‐CD80/CD86, triggering co‐inhibitory signaling. Contrastingly, in ATLL and other peripheral T‐cell lymphomas, CD28 undergoes copy number variations and activating mutations, leading to increased expression. This elevated CD28 expression results in the conversion of CTLA‐4‐mediated co‐inhibitory signals into CD28‐mediated co‐stimulatory signals, thereby fostering T‐cell proliferation and differentiation.^14^

Tumor‐associated macrophages (TAM) are categorized as M1 and M2 subtypes, with ATLL predominantly exhibiting M2 macrophages that impede anti‐tumor immunity. Additionally, TAMs release cytokines and chemokines that facilitate tumor proliferation.^15^ Signal Regulatory Protein α (SIRPα) is expressed in macrophages, while CD47 is a common marker on human cells. Their binding, known as the “don't eat me” signal, inhibits phagocytosis. Some tumor cells exhibit CD47 and/or SIRPα overexpression, utilizing the “don't eat me” signaling to evade immune response and facilitate tumor invasion. Analysis revealed no association between CD47 expression and prognosis, while stromal expression of SIRPα was identified as a positive prognostic factor.^16^

CC Chemokine Receptor 4 (CCR4) is also present in ATLL cells, making them vulnerable to anti‐CCR4 monoclonal antibody therapy. Among ATLL chemokines and their receptors, CC‐Ligand 18 (CCL18) is notably upregulated, while CX3C Chemokine Receptor 1 (CX3CR1) is downregulated, although neither is expressed on ATLL cells but on stromal cells. CCL18 acts as a marker for M2 macrophages and is linked primarily with monocytes, macrophages, and DCs. Its upregulation signifies ATLL immune evasion facilitated by TAMs.^17^ Conversely, CX3CR1 expressed on cytotoxic lymphocytes plays a vital role in pathogen and cancer cell elimination. The downregulation of CX3CR1 in ATLL indicates suppressed cytotoxic T‐cell functionality (Table 2).

**TABLE 2:** Immunologic alterations associated with HTLV‐1.

Markers	Changes	Effects
FOXP3+ Tregs		Conversion of naïve CD4+ T cells to the Treg phenotype. More susceptible to severe infections and Epstein–Barr virus infection
MHC I or β2M	Loss	Induces immune escape from CD8+ tumor‐infiltrating lymphocytes (TILs)
MHC II	Loss	Leading to immune escape from the attack of CD4+ TILs
CD58	Gene mutation or deletion	Tumor immune escape
PD‐L1 on ATLL cells binds to PD‐1 on T cells	Disruption of the 3′‐untranslated region(3′‐UTR) of the PD‐L1 gene	Immune escape through PD‐L1 overexpression
Tim‐3 binds to GAL9		Promotes immune escape and exhaustion of T‐cells in tumors
OX40/OX40L	HTLV‐1‐encoded oncoprotein Tax upregulates OX40 gene expression	Induces the proliferation and survival of T‐cells and their cytokine production. Suppress ATLL cells that usually demonstrate Treg phenotypes
Tumor‐associated macrophages (TAMs)		Tams exhibit an M2 phenotype and suppress anti‐tumor immunity. Tams also produce cytokines and chemokines to promote proliferation, angiogenesis, and metastasis of tumors
CD47/SIRPα	Overexpression	Promotes immune escape and tumor invasion, and is associated with a poor prognosis in some malignancies

#### 3.1.2 Impact of HTLV‐1 on immunotherapy

Approximately 27% of ATLL cases exhibit structural variants in the PD‐L1 gene, resulting in the destabilization of the 3′‐untranslated region (UTR). This destabilization leads to the stabilization and increased abundance of PD‐L1 transcripts, resulting in immune escape facilitated by PD‐L1 overexpression. The potential correlation between treatment with PD‐1 inhibitors and clinical staging of ATLL is a subject of investigation, and the association of PD‐1 blockade therapy with MHC class II expression in ATLL remains a topic of debate. The impact of immune checkpoint‐targeted therapies, including PD‐1 treatment, extends to TILs, ATLL cells, and stromal cells. Given that a majority of HTLV‐1‐infected T cells express CCR4, considered the preferred target for continual viral transmission, CCR4 emerges as a promising candidate for immunotherapy in ATLL.^18^ Mogamulizumab, a fully humanized and glycoengineered monoclonal anti‐CCR4 antibody, is currently chosen for the treatment of ATLL.

### 3.2 HPV

HPV, categorized into high‐, intermediate‐, and low‐risk types, is one of the most common sexually transmitted viruses, with high‐risk types being most carcinogenic. HPV‐induced cancers primarily affect females, with cervical cancer being the most common, but they can also lead to vaginal, penile, oral, anal, and skin cancers.^19^ Cervical cancer is predominantly linked to persistent high‐risk HPV infection, serving as a significant risk factor. Over 90% of cervical cancer cases are associated with high‐risk HPV infection. Following epithelial damage, HPV can infect the basal cell layer of keratin‐forming cells, with most individuals able to clear the infection completely. However, high‐risk HPV types such as 16 and 18 are more likely to persist and integrate into the host's genome.

#### 3.2.1 Impact of HPV on the tumor immune microenvironment

High‐risk HPV‐infected individuals exhibit significantly elevated levels of IL‐4 and decreased levels of IFN‐γ. Th2 cells primarily release IL‐4, promoting Th2 cell proliferation while hindering Th1 cell proliferation. Conversely, Th1 cells predominantly secrete IFN‐γ, fostering Th1 cell proliferation while impeding Th2 cell proliferation. Consequently, there is a Th1/Th2 imbalance in high‐risk HPV‐infected patients, leading to a substantial reduction in the body's antiviral function, hindering virus clearance, and increasing susceptibility to persistent HPV infection. TILs are enriched in HPV‐positive tumors, with CD4+ and CD8+ T cells showing responsiveness to peptides from all HPV genes. The level of activated T cells increases with tumor progression stage and decreases post‐treatment, indicating that the quantity of Cytotoxic T Lymphocytes (CTLs) induced by HPV antigens is dependent on the tumor burden, the primary source of antigens.^20^ Noteworthy pathways involved in HPV immune evasion and regulation within the TME include the downregulation of MHC molecules to impede cytotoxic T and NK cell activation, modulation of CD4+ T cell activation, suppressive role of regulatory T cells, regulation of the cGas‐STING pathway, and modulation of PD1/PD‐L1 immune checkpoints.

MHC class I plays a pivotal role in the eradication of virally infected and transformed cells. High‐risk HPV‐E5 can downregulate MHC class I protein expression,^21^ which hinders NK cell activation in clearing virus‐infected cells. This mechanism is considered oncogenic in cervical cancer, with some studies suggesting HPV‐E5's significant role in HPV immune evasion. Oropharyngeal cancers that are HPV‐positive demonstrate higher CD56+ cell infiltration compared to HPV‐negative squamous cell carcinomas of the head and neck, with the level of CD56+ infiltration correlating with prognosis.^22^ The development of HPV‐associated malignant tumors is linked to CD4+ T cell depletion, impacting the ability of CD8+ T cells to mount an effective immune response to HPV‐E7, crucial for tumor development and maintenance. The phenotype, rather than the sheer quantity, of CD4+ T cells is essential for effective anti‐HPV immunity. Foxp3+ regulatory T cells (Tregs), a CD4+ T cell subset, play a role in inhibiting CD8+ and CD4+ cells in the TME, restricting anti‐tumor immunity through downregulation of effector T cell induction and proliferation.^23^

The cGas‐STING pathway, a conserved antiviral pathway for DNA virus detection, exhibits heightened expression in HPV‐infected cells compared to uninfected cells. The HPV 18 E7 protein directly inhibits STING activity, downregulating STING through the induction of type I IFN genes.^24^ STING modulation appears to influence cetuximab‐mediated NK cell activation and tumor regression,^25^ suggesting a potential role of STING agonists in enhancing immune‐targeted therapeutic effects. Further preclinical and clinical studies are needed to establish the role of STING in HPV‐associated malignant tumors. TILs are more prominent in HPV‐positive tumors than in HPV‐negative counterparts, with several studies indicating that HPV positivity amplifies PD‐L1 levels in both tumor and infiltrating immune cells. PD‐1 expression on TILs does not impact survival, as PD‐L1 expression in cervical cancer acts as a poor prognostic factor, potentially secondary to cytotoxic T cell suppression.^26^, ^27^ In contrast to HPV‐negative tumors, HPV‐positive cancers showcase distinctive B‐cell‐associated characteristics, characterized by an increased proportion of germinal center B cells in HPV‐positive tumors and relatively fewer B cells in HPV‐negative tumors. These B cells within the TME are enriched with HPV‐specific antibody‐producing cells, contributing to inflammation.^28^

#### 3.2.2 Impact of HPV on immunotherapy

The prolonged existence of HPV‐associated tumor cells within the host, resulting in persistent host infection, has enabled the acquisition of numerous immune evasion strategies. This poses a significant obstacle to effectively treating HPV‐associated tumors with immunotherapy. The presence of HPV E7 cells demonstrates a notable correlation with PD‐L1 expression on tumor surfaces, enhancing recruitment of TILs and elevating IFN‐γ secretion, thus inducing dysfunction in CD8+ cells. HPV‐altered cells manipulate the local immune milieu to suppress effector immune responses through various mechanisms, such as triggering immunomodulatory cytokine release from fibroblasts^29^ and promoting the emergence of local innate M2 macrophages and antigen‐specific regulatory T cells.^30^ These factors present formidable challenges for successful immunotherapy targeting HPV‐related proteins in naturally occurring HPV‐associated malignancies in humans. Some immunomodulatory effects observed in HPV‐associated epithelial tumors may be attributed to epithelial cell proliferation rather than viral protein expression.^31^ Current immunotherapeutic strategies encompass adoptive T‐cell therapy, engineered TCR T‐cells, and PD‐1 and CTLA‐4 checkpoint blockade for cervical cancer intervention. Nonetheless, only pembrolizumab has garnered approval for clinical use in advanced cervical cancer, highlighting the gaps in existing immunotherapies for long‐term effects of chronic HPV infection.

### 3.3 HBV and HCV

HBV and HCV stand as the primary causes of liver cancer and mortality on a global scale. Approximately 257 million individuals worldwide are afflicted with chronic HBV infection, while 71 million grapples with chronic HCV infection.^32^ Since hepatitis B vaccine has been widely promoted in China in 1992, emerging as a pivotal tool in the control and prevention of hepatitis B and is extensively utilized in numerous countries. Regrettably, there is currently no effective vaccine available to prevent hepatitis C.

In individuals with chronic hepatitis B, the intrahepatic microenvironment can impede the effectiveness of immunotherapy. Various cytokines, including TNF, INF‐α, IFN‐γ, and IL‐1β, exhibit anti‐HBV replication properties and direct antiviral effects, yet their efficacy in patients with chronic hepatitis remains uncertain.^33^ Elevated intrahepatic SOCS3 levels, a negative regulator of cytokine signaling, were found to influence the effectiveness of IFNα in HCV patients. Notably, such levels were elevated in individuals with chronic hepatotropic viruses as well as in groundhog mice, concluding that cytokine efficacy may not be as marked as observed in experimental models of patients with chronic hepatitis B.^34^ The loss or decline in CD8+ T cell function in HCV infection contributes to viral persistence and immune evasion.^35^ Moreover, the ability of HCV NS3/4A to deactivate two host signaling pathways in response to HCV pathogens represents another evasion strategy employed by HCV.^36^

#### 3.3.1 Effects of HBV and HCV on the tumor immune microenvironment

HBV's sustainable replication capability hinges on its inhibitory impact on innate immune cells like natural killer (NK) cells and IFN‐1‐type responses.^37^ Immunosuppressants are present in both innate and adaptive immune cells, leading to deficiencies in effective immune responses against HBV. These deficiencies include increased regulatory T cells (Tregs), CD4+ T cell depletion, upregulation of programmed death receptor‐1 and its ligands (PD‐1/PD‐L1), inhibition of cytotoxic T‐lymphocyte‐associated protein 4 (CTLA‐4), inhibitory pathways of CTLA‐4, dendritic cell (DC) dysfunction, and decreased Toll‐like receptor (TLR) expression.^38^ Furthermore, the covalently closed circular DNA (cccDNA) created by the HBV gene enables viral concealment, contributing to prolonged HBV presence in the host and hindering its elimination.

Immunotherapeutic approaches aiming to enhance HBV‐specific T‐cell responses encounter obstacles due to the infiltration of inflammatory factors in patients with chronic active hepatitis, such as TNF released by inflammatory monocytes, escalating faster in advanced stages of chronic viral hepatitis B (CHB).^39^ Liver enrichment of Tregs in patients with chronic hepatitis B not only represses the inflammatory response but also dampens HBV‐specific T cells. Arginine, an essential amino acid for virus‐specific T cell function, faces depletion in the liver due to the release of arginase by deceased hepatocytes, affecting T cell function.^40^ The enduring presence of soluble forms of HBsAg and HBeAg, distinctive in HBV infection, carries implications for chronic hepatitis B immunotherapy. The effect of these viral proteins on host immunity remains unsettled, with some studies suggesting that persistent exposure to HBsAg and HBeAg may undermine the number and function of myeloid cells and plasma cell‐like DCs,^41^ hinder TLR‐mediated cytokine production, regulate the surface expression of the innate immunity receptor TLR2,^42^ and inhibit antigen presentation, thereby altering T cell cytokine production and HBV‐specific T cell generation.^43^, ^44^ Notably, the persistence of HBsAg has been identified to disrupt HBsAg‐specific B cell functionality, leading to defective antibody production, atypical memory phenotypes, and suppression of receptor upregulation.^44^, ^45^

HCV's oncogenic impact on liver parenchyma primarily results from HCV‐related proteins such as core proteins, NS2, NS3, and NS5A, which impede apoptosis and spur cell proliferation.^46^ Numerous studies indicate that HCV infection in hepatocytes impairs antiviral host immunity by modulating the expression of immunoregulatory molecules. Most of the pathways (about 95%) implicated in the immune response to HCV‐related liver cancer are suppressed. These pathways encompass genes related to both innate and adaptive immunity, especially genes associated with T‐cell‐mediated immune responses. During chronic HCV infection, NK cells undergo phenotypic and functional modifications, impacting B cell immune function. Furthermore, there is a decline in NF‐κB signaling activation in HCV‐associated hepatocytes, with HCV NS5A elevating interferon‐induced STAT1 signaling degradation. Profound dysregulation of T‐cell response and activation pathways and suppression of T‐cell‐related genes are observed in HCV‐associated hepatocytes, though not in HCV‐associated tumors.^47^

Both HBV and HCV modulate the tumor immune microenvironment of hepatocellular carcinoma (HCC) by impairing T‐cell functionality, leading to CD8+ and CD4+ T lymphocyte dysfunction. HCV infection induces a notable upsurge in TGF‐β and over‐regulation of PD‐L1/PD‐1, dampening the T‐cell response and fostering immunosuppression in HCC. TGF‐β secretion exerts a negative impact on T effector cells, while the PD‐1/PD‐L1 axis significantly contributes to cancer immune evasion, tumor severity, and poor prognosis.^48^

#### 3.3.2 Impact of HBV and HCV on immunotherapy

There is a mounting body of evidence indicating that cancer stem cells (CSC) possess the ability to suppress the immune microenvironment in HCC through various intrinsic and extrinsic mechanisms, ultimately resulting in immune evasion. The activation of immune‐related pathways specific to CSCs, the down‐regulation of antigen processing (TAP) and MHC molecules, as well as elevated levels of CD47 and PD‐L1 expression collectively contribute to immune escape in HCC.^49^ CTLA‐4 functions by inhibiting T‐cell activation through the suppression of the costimulatory ligands CD80 and CD86. Various external factors such as alcoholic or nonalcoholic steatohepatitis, hypoxic conditions, and abnormal angiogenesis also play a role in this process. Immunotherapeutic strategies currently utilized for targeting HCC CSCs primarily encompass antibody immunotherapy based on CSC markers, immune checkpoint inhibitors, antiangiogenic therapy, CAR‐T/TCR‐T cell therapy, NK cell‐based cancer immunotherapy, and DC vaccines. Despite advancements, there remain hurdles impeding their optimal efficacy. Given that CSCs represent a rare subset within tumor tissues, exclusively targeting CSCs may not suffice in achieving complete tumor eradication. Hence, combining CSC‐targeted immunotherapy with established cancer treatments such as chemotherapy, radiotherapy, anti‐angiogenic therapy, and checkpoint inhibitors could prove to be a more potent approach in combating HCC.

### 3.4 EBV

EBV, also known as human herpesvirus 4 (HHV‐4), is a member of the herpesvirus C family and infects over 95% of the global population, mainly through saliva transmission. EBV establishes lifelong infection by infecting B cells and residing in memory B cells.^50^ In carriers, viral loads are low (around 0.01% of B lymphocytes) and primary infection is usually asymptomatic. However, genetic mutations or factors like immunosuppression, HIV, and high‐salt diets can contribute to EBV‐related cancers. In these cancers, the virus is present in every tumor cell, expressing latent viral genes and showing a clonal or oligoclonal pattern. Cancer development in EBV cases typically takes years. The EBNA1 protein plays a critical role in maintaining the stability of the viral genome throughout cell replication, essential for ensuring the persistence of the viral genome.

EBV exhibits four latency modes (0, I, II, and III), with variations in latency mode observed across different diseases. To evade detection by the host immune system, EBV significantly restricts the expression of viral proteins during latency. Key genes associated with latency include EBNAs (Epstein–Barr nuclear antigens), LMPs (latent membrane proteins), and certain non‐coding transcripts. In latency 0, no EBV protein is expressed; latency I involves solely EBNA1 expression; in latency II, expression is confined to EBNA1, LMP1, and LMP2; and in latency III cells, EBNA1‐6, LMP1, and LMP2 are expressed. These latency patterns are evident in various EBV‐associated malignancies, such as post‐transplantation lymphoma (latency III), Hodgkin's disease and nasopharyngeal carcinoma (both latency II), and Burkitt's lymphoma (latency I).^51^

#### 3.4.1 Effect of EBV on the tumor immune microenvironment

EBV evades the host's innate immunity through several mechanisms: reducing TLR expression, disrupting host IRF signaling and type I interferon production, interfering with NF‐κB and inflammatory pathways, and targeting effector molecules of innate immunity, such as cytokine colony‐stimulating factor 1. To evade recognition and elimination by antiviral CD8+ T cells, EBV encodes a minimum of three proteins that hinder antigen presentation. These include BGLF5, which generally interferes, BNLF2a, which inhibits TAP, and BILF1, which downregulates surface HLA I classes. These viral proteins are expressed early in the EBV replication cycle, thereby preventing CD8+ T cell detection. Additionally, the gp42 protein binds to HLA class II molecules on B cells, disrupting the interaction between the T cell receptor (TCR) and HLA class II molecules, which results in the inhibition of CD4+ T cell activation. Moreover, the viral proteins gH/gL stabilize and enhance the expression of gp42. Furthermore, EBV BZLF1 counteracts innate effector molecules by down‐regulating TNF‐α and IFN‐γ receptors while inducing the inhibitor of cytokine signaling 3 (SOCS3). SOCS3 inhibits JAK/STAT signaling, leading to a refractory state towards type I IFNs and reducing IFNα production by monocytes.

#### 3.4.2 Impact of EBV on immunotherapy

Cytotoxic T lymphocytes (CTLs) play a crucial role in host immune regulation through the secretion of various cytokines, targeting the killing of virus‐infected cells and tumor cells, and serving as a vital defense line against viruses and tumors. Currently, EBV‐specific CTLs can be generated from capturing B‐lymphoblastoid cell lines (B‐LCLs), DCs, and interferon‐gamma (IFN‐γ). Studies in animals have demonstrated the effectiveness and safety of EBV‐CTLs against EBV‐associated lymphomas. However, despite these promising outcomes, the clinical use of EBV‐CTLs is limited due to various constraints. Chimeric antigen receptor T‐cell therapy (CAR‐T) is an emerging immunotherapeutic approach that redirects activated T cells towards specific targets by modulating their natural TCR and co‐stimulatory pathways through tumor‐specific CAR gene modifications.^52^ CAR‐transduced CTLs have shown potential in targeting EBV‐infected B cells and tumor cells, which could mitigate the risk of lymphoproliferative disorders and enhance the prognosis of transplant patients.^53^ Nonetheless, Hodgkin's lymphoma cells lack the expression of dominant EBV antigens, leading to immune evasion from CTL‐based therapies, rendering them potentially ineffective.

### 3.5 KSHV/HHV‐8

KSHV, also known as human herpesvirus 8, is the causative agent of Kaposi's sarcoma (KS) and is linked to multicentric Castleman's disease (KSHV‐MCD) and primary effusion lymphoma (PEL).^54^ KSHV and EBV have the ability to infect and transform the same B cells typically present in PEL. The viral protein ORF73, known as latency‐associated nuclear antigen (LANA), is universally expressed in all KSHV‐infected cells and serves as a diagnostic marker for KSHV‐related diseases. LANA, along with various viral microRNAs, plays a crucial role in maintaining viral episomes, evading the host's immune response, and promoting cell survival and proliferation, facilitating the persistence of the virus.

#### 3.5.1 Effect of KSHV on the tumor immune microenvironment

Kaposi's Sarcoma (KS) is the most common tumor associated with HIV‐1 infection. It shows a higher incidence in other immunosuppressed individuals, such as post‐organ transplantation recipients, as well as populations with a specific susceptibility to KSHV infection. KSHV has developed evasion strategies against both innate and adaptive immunity, promoting cell proliferation and preventing apoptosis in infected cells, ultimately leading to tumorigenesis. While virus‐infected cells can be targeted and eliminated by CD8+ T cells and NK cells, certain KSHV proteins, like KSHV K3 and KSHV K5, recruit supportive helper cell populations, creating an inflammatory microenvironment and impairing the functions of T and NK cells. KSHV Replication and Transcription Activator (RTA) facilitates the proteasomal degradation of MHC‐II and upregulates membrane‐associated RING‐CH, an MHC‐II antagonist, to evade immune modulation mediated by CD4+ T cells.^55^ Additionally, KSHV Latency‐Associated Nuclear Antigen (LANA) contributes to the downregulation of MHC‐II expression. The virus also induces an inflammatory cytokine environment, particularly in the presence of KSHV‐associated malignancies, to enable the growth and proliferation of infected cells while undermining components of the host immune system. The primary infection of monocytes with KSHV triggers the secretion of pro‐inflammatory factors IL‐1α, IL‐1β, and IL‐6. Cells recognize viruses through the binding of pathogen‐associated molecular patterns (PAMP) to pattern recognition receptors (PRR), with TLRs being the most recognized PRRs. Membrane‐bound TLRs 1, 2, 4, 5, 6, and 10 detect structural components of invasive pathogens like viral glycoproteins, whereas vesicular TLRs 3, 7, 8, and 9 present inside the cell recognize pathogenic DNA or RNA.^56^

#### 3.5.2 Impact of KSHV on immunotherapy

PD‐L1 is commonly overexpressed in various tumor types and acts as a suppressor molecule; KSHV infection enhances PD‐L1 expression in monocytes, potentially leading to immune evasion.^57^ CTLA‐4, another prominent target for immunotherapy, functions by inhibiting B7‐mediated T‐cell co‐stimulation.^58^ KSHV K3 prompts the downregulation of solubilized MHC‐I, while lenalidomide restores MHC‐I expression.^59^ Diseases linked to KSHV often exhibit elevated IL‐6 levels, prompting a consideration of anti‐IL‐6 antibodies for novel therapeutic approaches.

### 3.6 MCPyV

MCPyV is a prevalent cutaneous polyomavirus that plays a pivotal role in the carcinogenesis of Merkel cell carcinoma (MCC). Typically acquired during childhood without manifesting any recognizable signs or symptoms, MCPyV can be found in the skin of the majority of healthy individuals. Although most people come into contact with MCPyV during their lifetimes, only a minority develop MCC. MCC, a rare neuroendocrine skin carcinoma, is primarily linked to MCPyV infection.^60^ Alongside MCPyV, ultraviolet radiation (UVR) serves as a significant pathogenic factor in MCC by inducing mutations in the MCPyV LT antigen and upregulating the ST antigen, leading to local immunosuppression and contributing to viral carcinogenesis. MCC tumors impede T cell migration to inflamed areas by disrupting the interaction between T cell surface receptors and tumor endothelial cells. They accomplish this by altering gene expression, which results in reduced MICA/MICB and MHC levels critical for NK/T cell‐mediated lysis of tumor cells. Furthermore, MCC tumors enhance the expression of inhibitory receptors on immune cells, deactivate effector T cells approaching the tumor site by expressing surface receptors, notably PD‐L1, and recruit regulatory T cells (Tregs) to the inflamed area, thereby suppressing the immune response. Through these evasion mechanisms, MCC manages to circumvent immune surveillance, ensuring its persistence in the host organism.^61^

#### 3.6.1 Effect of MCPyV on the tumor immune microenvironment

It has been demonstrated that various pro‐inflammatory molecules, particularly IL‐8, CXCL1, IL‐6, IL‐1β, MMP1, and CXCL6, exhibit significant upregulation in MCC tumors.^62^ Ultraviolet radiation (UVR) is linked to the recruitment of regulatory T cells, which can exert direct damage on APCs or hinder APC function through the release of cytokines by mast cells, such as IL‐10 and TNF‐α. MCC tumor cells are believed to create a local immunosuppressive milieu by overproducing immunosuppressive elements like TGF‐β, Fas‐L, IL‐10, or T‐cell response inhibitors such as galactoglucan lectin‐1 and indoleamine 2,3‐dioxygenase (IDO). Additionally, tumors may disrupt the activated STAT3 pathway to impede pro‐inflammatory danger signals, leading to impaired dendritic cell maturation, or diminish NKG2D receptor expression on immune effector cells by secreting soluble MIC NKG2D ligands, thereby dampening lymphocyte‐mediated cytotoxicity. Tumor cells can also facilitate the production, activation, or function of immunosuppressive cells,^63^ such as CD4 + CD25+ regulatory T‐cells (T‐regs) or myeloid‐derived suppressor cells (MDSCs). MCC attracts Tregs to inflamed regions while hindering T‐cell migration to such regions and reducing MICA/MICB and MHC expression through gene modulation. High viral loads lead to more severe CD8+ T cell exhaustion. The interaction of PD‐1 on T cells with its B7H1 or PDL‐1 ligands serves as a crucial mechanism for T cell depletion and can be targeted for therapeutic interventions. Both viral and UV‐mediated MCC tumors exhibit loss of MHC‐1 and β2‐microglobulin (β2M), a common immune evasion mechanism observed in various cancers.^64^ In MCC tumors, cell surface expression of MHC‐1 is diminished, and decreased gene expression of MHC‐1 associated with β2M has been noted in MCC cell lines.^65^

#### 3.6.2 Impact of MCPyV on immunotherapy

Immunotherapy has focused on the PD‐1/PD‐L1 immune checkpoint pathway as a crucial target for reinvigorating the immune response against diverse cancer types.^66^ The potential effectiveness of targeting this pathway for MCC is supported by various indications: MCC has been recognized as an immunogenic cancer, evidenced by the higher incidence and poorer prognosis in immunosuppressed individuals. Additionally, there is an immune response to the MCPyV T‐antigen in the blood of MCC patients.^67^ Some MCCs exhibit enrichment of tumor‐infiltrating T‐cells, whether specific or nonspecific to the MCPyV protein.^68^ Immunotherapy shows promise in treating advanced MCC, surpassing the efficacy of previous treatments; however, a significant portion of advanced MCC cases do not respond to PD‐1/PD‐L1 inhibitors. Consequently, ongoing clinical trials explore immune checkpoint inhibitors for MCC, including combinations with CTLA‐4 inhibitors, T‐cell or NK cell transfers, or innovative therapeutic agents. MCC cells may lack expression of MHC molecules, compromising both adaptive and innate immune responses.^69^ Reversal of MHC class I downregulation can be achieved through class I interferon therapy or epigenetic modifications. However, interferon therapy may inhibit the MCPyV T antigen, a critical immunogenic epitope, potentially reducing MCC cell susceptibility to immune detection. Preclinical models have shown that epigenetic modulation of HLA class I expression does not interfere with T antigen expression; this treatment reactivates molecules that engage the innate immune response. Ongoing clinical trials in MCC combine multiple therapeutic strategies with immune checkpoint inhibitors to counteract or evade immune escape mechanisms efficiently.^70^

**FIGURE 1:**
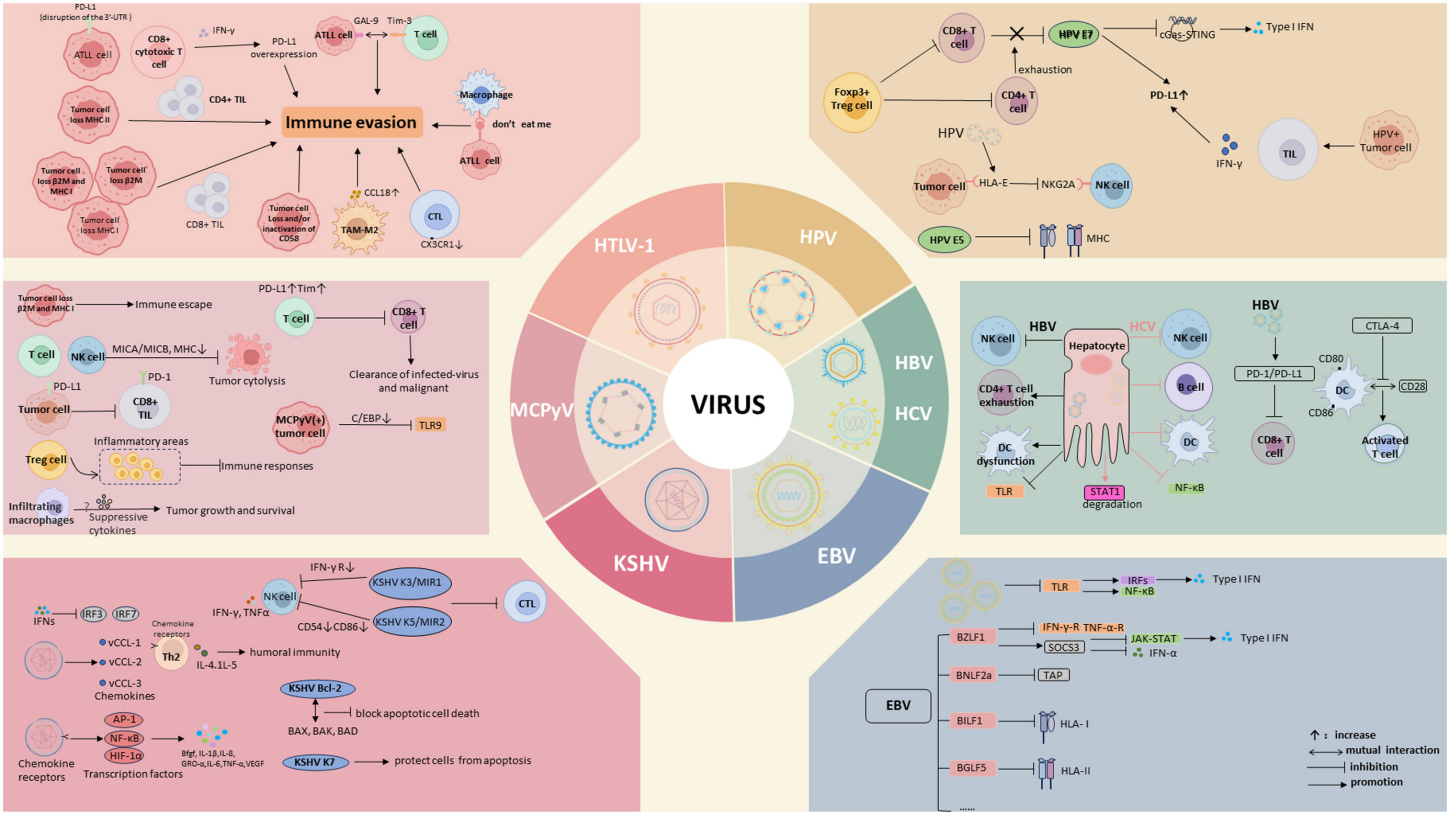
Impact of Seven Oncogenic Viruses on the Tumor Immune Microenvironment. The effects of HTLV‐1, HPV, HBV, HCV, EBV, KSHV, MCPyV and HTLV‐1 on the immune microenvironment in related tumors are summarized. PD‐L1, Programmed death ligand 1; PD‐1, Programmed cell death protein 1; TILs, Tumor‐infiltrating lymphocytes; SIRPα, Signal‐regulated protein α; don't eat me, Cluster of Differentiation 47 (CD47), releases the “don't eat me” signal when it binds to its receptor, SIRPα; GAL‐9, Galectin‐9; TIM‐3, T cell immunoglobulin domain and mucin domain‐3; TAPs, Transporter associated with antigen processing; IFN, Interferon; MICA/MICB, MHC class I chain‐related molecules A/B; UTR, Untranslated region; TLR, Toll‐like receptor; IRF, Interferon regulatory factor.

For a summary of the altered immune microenvironment of seven cancer‐related viruses above refer to Figure 1.

### 3.7 Helicobacter pylori

*H. pylori* is a Gram‐negative, spiral‐shaped bacterium linked to various gastrointestinal disorders, including gastritis, peptic ulcers, and GC. Recognized as a class I carcinogen by the International Agency for Research on Cancer (IARC), many infections are asymptomatic.^71^ The majority of GCs worldwide are attributed to the inflammatory damage induced by *H. pylori*. *H. pylori* secrete diverse virulence factors that can disrupt host cell signaling pathways and promote tumorigenesis by lowering the tumor transformation threshold. Among these factors, CagA (cytotoxin‐associated gene A) and its pathogenicity island (Cag PAI), along with VacA (vacuolar cytotoxin A), are considered key virulence factors.^72^ The activation of NF‐κB and the upregulation of IL‐8 in gastric epithelial cells are deemed pivotal mechanisms in the development of *H. pylori*‐induced chronic inflammation and GC. Ultimately, the risk of GC development hinges on a complex interplay of factors, including strain‐specific virulence factors of *H. pylori*, host genetics, environmental influences like diet, and the turnover of stem cell populations and microbiome.^73^

#### 3.7.1 Impact of *H. pylori* on the tumor immune microenvironment

**FIGURE 2:**
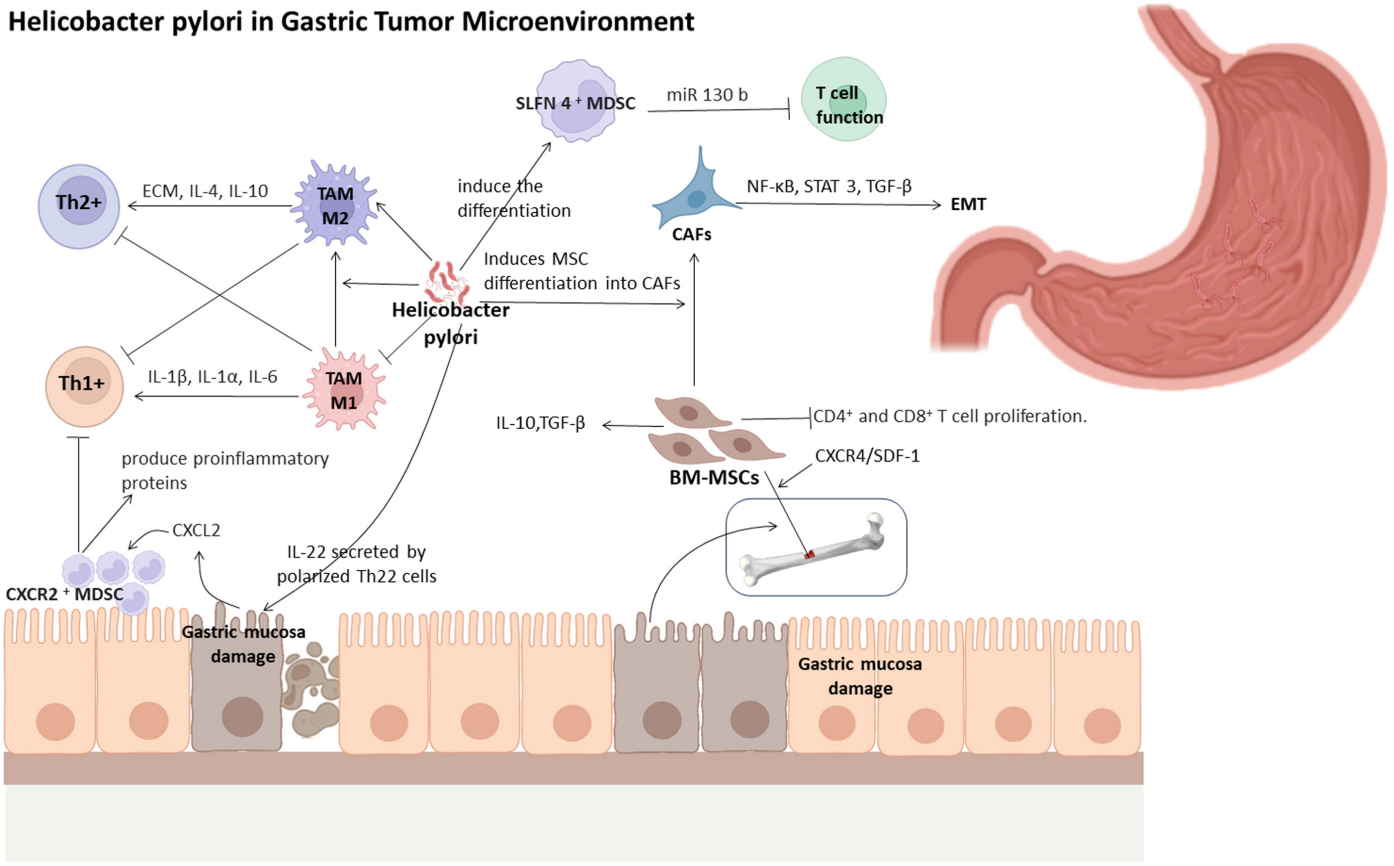
The Influence of Helicobacter pylori (*H. pylori*) on the Microenvironment of Gastric Tumors. *H. pylori*'s association with tumor‐associated macrophages (TAMs) influences immune responses and tumor progression. M1 macrophages produce pro‐inflammatory cytokines for immune defense, while M2 macrophages contribute to wound healing and immune moderation. *H. pylori* can shift macrophage balance, potentially aiding tumor growth. Bone marrow mesenchymal stem cells (BM‐MSCs) migrate to the stomach, responding to mucosal damage cues, aiding in tissue repair through secretion and guided motility. When introduced into *H. pylori*‐infected mice, BM‐MSCs increase anti‐inflammatory secretions and inhibit T cell proliferation. Infection prompts MSCs to become cancer‐associated fibroblasts. *H. pylori* influences MDSCs and Schlafen 4+ MDSCs, resulting in altered immune responses. MDSCs suppress gastritis and T cell function. Additionally, *H. pylori* stimulates the secretion of IL‐22, promoting an inflammatory response that contributes to gastritis development.

*H. pylori* exerts influence on various cell types within the immune microenvironment of gastric tumors, including tumor‐associated macrophages (TAMs), bone marrow‐derived mesenchymal stem cells (BM‐MSCs), cancer‐associated fibroblasts (CAFs), MDSCs, and other stromal cells. Human adipose‐derived mesenchymal stem cells (hA‐MSCs) also enhance tumor invasion and metastasis.^74^ BM‐MSCs can decrease the proportion of IFN‐γ‐producing T cells, thereby hindering CD4+ and CD8+ T cell proliferation. *H. pylori* infection upregulates THBS4 (platelet‐responsive bromodomain 4) expression in BM‐MSCs, promoting GC angiogenesis. Moreover, *H. pylori* infection leads to the differentiation of MSCs into CAFs, where fibroblast activation protein (FAP)‐positive CAFs support the survival, proliferation, and migration of GC cell lines while suppressing T cell activity.^75^ *H. pylori* infection also elevates hepatocellular carcinoma‐derived growth factor (HDGF) expression, boosts BM‐MSC recruitment, and enhances tumor cell proliferation, invasiveness, and metastasis.^76^ *H. pylori*‐induced CAFs drive epithelial‐mesenchymal transition (EMT), furthering cancer progression and metastasis.^77^ Additionally, MDSCs can promote immunosuppressive pathways and deplete essential metabolites for T cell function. *H. pylori* induces the differentiation of myeloid differentiation factor Schlafen 4 (SLFN4) MDSCs, which impede *H. pylori*‐induced gastritis responses and suppress T cell functionality.^78^ Furthermore, *H. pylori* stimulates interferon‐α secretion from plasmacytoid DCs via the TLR9‐MyD88‐IRF7 pathway. Several markers, including MiR130b, apoptosis signal‐regulated kinase 1 (ASK1), interleukin 22 (IL‐22), programmed death ligand 1 (PD‐L1), and Kruppel‐like factor 4 (KLF4), play critical roles in mediating the interplay between *H. pylori* and MDSCs. *H. pylori* infection downregulates KLF4 expression through inducing CXCL8 expression, leading to heightened CXCL8 levels that facilitate MDSCs recruitment to tumors.^79^ This surge in CXCL8 expression fosters tumor growth, shifts the microenvironment towards immunosuppression, and fortifies resistance against immune responses.^80^ The effect of *H. pylori* to gastric TME is referred to Figure 2.

#### 3.7.2 Impact of *H. pylori* on immunotherapy

*H. pylori* infection leads to the induction of Programmed Death‐Ligand 1 (PD‐L1) expression and myeloid‐derived suppressor cell (MDSC) infiltration, facilitating immune evasion mechanisms. HP infection triggers Hedgehog (HH) signaling activation, which in turn upregulates PD‐L1 expression in GC cells and promotes tumor cell proliferation, thereby contributing to cancer cell resistance to immunotherapy.^81^ Furthermore, *H. pylori* and its virulence factors, such as CagA, VacA, blood group antigen‐binding adhesin gene (BabA), and Helicobacter pylori neutrophil‐activating protein (HP‐NAP), can serve as antigens or adjuvants to enhance tumor immunity. The presence of autoantibodies during antigen processing and presentation, along with subsequent T‐cell activation and proliferation, enhances the host immune response, leading to cancer cell death and potential inhibition of metastasis. Additionally, HP‐DNA vaccines encoding CagA, VacA, and BabA fragments have been shown to elicit a shift from a Th1 to Th2 response in immunized BALB/c mice, resembling the immune profile of GC patients with chronic *H. pylori* infection. In vitro studies demonstrated that activated CD3+ T cells suppress the proliferation of human GC cells, and in vivo experiments revealed that infusion of CD3+ T cells inhibits the growth of GC xenografts.^82^

HP‐NAP emerges as a significant virulence factor in *H. pylori* infection and colony formation, while also exhibiting protective properties. Functioning as a Toll‐like receptor‐2 (TLR2) agonist, HP‐NAP interacts with neutrophil TLR2.^83^ Moreover, HP‐NAP promotes dendritic cell (DC) maturation, induces Th1 polarization, and enhances the migration of mature DCs. By stimulating neutrophils and monocytes, HP‐NAP triggers the expression of interleukin‐12 (IL‐12) and interleukin‐23 (IL‐23), thereby skewing the antigen‐specific T‐cell response towards a Th1 phenotype characterized by the abundant expression of IFN‐γ and tumor necrosis factor‐alpha (TNF‐α).^84^ Vaccination with the *H. pylori*‐NAP A subunit (NapA) favors Th17 and Th1 polarization, establishing this vaccine as a potential anti‐*H. pylori* oral vaccine candidate and mucosal immunomodulator for anti‐tumor therapy.^85^ In conclusion, *H. pylori* and its virulence factors hold promise for personalized treatment approaches in the realm of tumor immunotherapy.

## 4 RECENT STUDIES ABOUT CARCINOGENIC PATHOGENS

Recent studies have revealed that certain indigenous microbes, normally found in the oral cavity or digestive tract, may foster tumorigenesis and progression when immune conditions are compromised. They achieve this by establishing a pro‐inflammatory immune milieu or engaging in intercellular interactions, which also can influence the efficacy of cancer treatments.

### 4.1 Fusobacterium nucleatum

*F. nucleatum*, an anaerobic Gram‐negative bacillus predominantly inhabiting the oral cavity, plays a crucial role in maintaining the normal oral microenvironment.^86^ It has been shown that *F. nucleatum* increases tumor multiplicity and selectively recruits tumor‐infiltrating myeloid cells in the ApcMin/+ mouse tumor model, which can promote tumor progression. Collectively, fusobacteria, generate a pro‐inflammatory microenvironment that is conducive for colorectal neoplasia progression. Investigations have revealed that oral *F. nucleatum* difficile can transit to the colon via the digestive tract, potentially disseminating hematogenously due to frequent gingival bleeding episodes.^87^ Noteworthy for its prevalence in colorectal, esophageal, pancreatic, and breast cancers, *F. nucleatum* accelerates tumor progression and metastasis, fosters a pro‐tumorigenic microenvironment, and impedes tumor‐infiltrating lymphocytes.^88^ *F. nucleatum* riggers the production of inflammatory mediators like IL‐6, IL‐8, and COX‐2 through the NF‐κB pathway, linking it to colorectal carcinogenesis. Excessive *F. nucleatum* presence correlates with poorer prognoses in these malignancies. While the prevalence of *F. nucleatum* infection in CRC is mounting, its direct involvement in tumorigenesis remains ambiguous.

### 4.2 Streptococcus anginosus and Peptostreptococcus anaerobius

Prof. Yu's work had recently revealed that Streptococcus anginosus (*S. anginosus*), are linked to gastritis and gastric tumors.^89^ *S. anginosus* was enriched in the gastric mucosa of patients with GC. TMPC, a surface protein of *S. anginosus*, interacts with the Annexin A2 (ANXA2) receptor of gastric epithelial cells, which mediates adhesion and colonization of *S. anginosus* and induces the activation of MAPK. In short, *S. anginosus* promotes gastric tumorigenesis through direct interactions with gastric epithelial cells through TMPC‐ANX2‐MAPK axis. However, the effect of *S. anginosus* on the immune microenvironment of GC and its relationship with other microorganisms such as *H. pylori* remains an area for further research.

Professor Yu's another remarkable work provides an in‐depth exploration of the impact of *P. anaerobius* on the tumorigenesis and therapeutic outcomes in CRC.^90^ *P. anaerobius* is an anaerobic bacterium selectively enriched in the fecal and mucosal microbiota of CRC patients. It not only promotes CRC cell proliferation but also triggers a pro‐inflammatory microenvironment for colorectal tumorigenesis. It directly interacts with integrin α2/β1, which is overexpressed in CRC cells, via the surface protein PCWBR2. The interaction of PCWBR2 with integrin α2/β1 promotes the adhesion of *P. anaerobius* and the initiation of an oncogenic PI3K‐Akt‐FAK cascade that promotes the proliferation of tumor cells, the remodeling of CRC cells, and promoting tumorigenesis. Recently, some studies have found that *P. anaerobius* mediate resistance to PD‐L1 inhibitors. Anti‐PD‐1 antibodies in the presence of *P. anaerobius* exert little antitumor effect. The bacterium not only attracts MDSCs to the tumor but also activates the immunosuppressive activity of MDSC by activating the Slamf4 receptor of MDSC through the secretion of lytc‐22 protein, which creates an immunosuppressive microenvironment conducive to CRC by recruiting MDSC and reducing the infiltration of functional T cells. Depletion of MDSC by Ly6G (Lymphocyte antigen 6 complex locus G6D) or targeting of integrin α2/β1 or Slamf4 receptors could alter the anti‐PD‐1 treatment resistance of *P. anaerobius*.

## 5 CONCLUSIONS AND PERSPECTIVES

The TME is pivotal in cancer development and progression, offering a promising avenue for identifying novel targets in cancer immunotherapy by investigating its multifaceted components and intricate interactions. The body's response to viruses and bacteria often triggers inflammation, establishing a link between chronic inflammation and the onset of various cancers like gastric, liver, and bladder cancers. Infectious agents like *H. pylori* are known to induce chronic inflammation, heightening the risk of cancer development.^91^ Key pathways implicated in inflammation‐driven carcinogenesis intersect at signal transducers and activators of transcription factor 3 (STAT3) and NF‐κB levels.^92^

The presence of specific pathogenic microorganisms significantly influences cancer treatment. For instance, *F. nucleatum*‐derived succinic acid causes tumor resistance to immunotherapy in CRC. *F. nucleatum* also promotes chemoresistance in CRC by modulating autophagy. Reducing a specific gut microbe in CRC patients may i mprove their response to chemotherapy and reduce cancer recurrence. Recently, a phage targeting tumor‐colonizing *F. nucleatum* has emerged,^93^ suggesting that future therapeutic strategies for various tumors may entail targeting *F. nucleatum*. Conversely, certain pathogenic microorganisms' metabolic byproducts can boost the host's immune response during infection. This highlights the importance of comprehending the pathogenic mechanisms of chronic microbial infections and utilizing targeted medications to eradicate these pathogens or their secretions, thereby potentially enhancing the body's receptiveness to cancer treatment.

In addition, cancer vaccines are anticipated to become the cornerstone of tumor therapy, aiming to address immunosuppression within the TME. Compared to traditional radiotherapy, cancer vaccine technology has shown significant advantages. Recent developments in novel cancer vaccines have instilled optimism among oncologists regarding the imminent transformative progress brought on by immunotherapy. On one hand, certain chronic infections, as we mentioned in this review, have been confirmed to be associated with specific types of tumors. These pathogens may promote tumor development through mechanisms such as chronic inflammation, direct cell transformation, or immune escape. Therefore, developing vaccines targeting these pathogens can serve as a strategy to prevent the occurrence of related tumors. For patients already infected with chronic pathogens, tumor vaccines may help enhance the immune system's ability to recognize and eliminate tumor cells. Furthermore, combining the treatment of chronic infections with tumor vaccine administration may enhance therapeutic outcomes. For instance, clearing chronic infections may aid in restoring immune system function, thereby boosting the effectiveness of tumor vaccines. However, on the other hand, chronic infections may impact the efficacy of tumor vaccines, as the presence of pathogens could exhaust the immune system, leading to immune suppression. Therefore, future research needs to consider how to optimize vaccine administration strategies to overcome the challenges posed by chronic infections.

In addition to discussing carcinogenic microorganisms in the text, the development of many tumors is also associated with chronic infections of certain microorganisms. For example, CT infection can act as a cofactor for HPV, promoting the progression to invasive cervical cancer (ICC). CT infections on cervix may lead to local immune dysregulation, favoring the persistence or progression of infections caused by high‐risk HPV types.^94^ Mycoplasma's colonization appears to be more common in prostate cancer patients.^95^ However, there is still insufficient evidence confirming a direct association between CT or Mycoplasma colonization or infection and cervical or prostate cancer. Additional studies are needed to investigate the correlations and underlying mechanisms linking these pathogens to tumors. Such investigations aim to mitigate tumor incidence and improve tumor treatment outcomes by preventing or eliminating these pathogen infections.

While immunotherapy has garnered significant attention in recent years, its effectiveness remains limited for numerous diseases. Despite extensive investigations into the immune escape mechanisms of some common carcinogenic pathogens discussed in this paper, some mechanisms still elude comprehension. Moreover, the efficacy of immunotherapy against various bacteria and viruses is suboptimal, with a lack of well‐defined therapeutic targets. The intricate nature of the components within the TME and their interactions poses significant challenges to immunotherapy research, warranting further exploration. We expect that continued research and development of immunotherapy will continue to revolutionize the field of cancer treatment and provide patients with more treatment options.

## AUTHOR CONTRIBUTIONS

**Si Chen:** Investigation (equal); methodology (equal); writing – original draft (equal); writing – review and editing (equal). **Caihong Yao:** Investigation (equal); resources (equal); visualization (equal); writing – original draft (equal). **Na Tian:** Investigation (equal); resources (equal). **Chunying Zhang:** Data curation (equal); resources (equal). **Yuemei Chen:** Data curation (equal); investigation (equal); resources (equal). **Xuting Wang:** Investigation (equal); resources (equal). **Yue Jiang:** Investigation (equal); resources (equal). **Tonghao Zhang:** Investigation (equal); software (equal). **Tingting Zeng:** Conceptualization (equal); funding acquisition (equal); project administration (equal); supervision (equal). **Yali Song:** Conceptualization (equal); funding acquisition (equal); project administration (equal); supervision (equal); writing – review and editing (equal).

## FUNDING INFORMATION

This work was supported by 1·3·5 project for disciplines of excellence, West China Hospital, Sichuan University (ZYJC21067), National Natural Science Foundation of China (82302632), the Natural Science Foundation of Sichuan Province (2022NSFSC1305 and 2022NSFSC0747), the Sichuan Science and Technology Program (2022YFS0214).

## CONFLICT OF INTEREST STATEMENT

The authors have no competing interests to disclose.

## Data Availability

The review was based entirely on previously published data.
